# Robotic-Assisted Solutions for Invasive Cardiology, Cardiac Surgery and Routine On-Ward Tasks: A Narrative Review

**DOI:** 10.3390/jcdd10090399

**Published:** 2023-09-18

**Authors:** George Koulaouzidis, Dafni Charisopoulou, Piotr Bomba, Jaroslaw Stachura, Pawel Gasior, Jan Harpula, John Zarifis, Wojciech Marlicz, Damian Hudziak, Tomasz Jadczyk

**Affiliations:** 1 Department of Biochemical Sciences, Pomeranian Medical University, 70-204 Szczecin, Poland; koulaou@yahoo.co.uk; 2 Pediatric Cardiology Department, Great Ormond Street Hospital, London WC1N 3JH, UK; dafnithess@yahoo.co.uk; 3 InBioSens, 31-351 Krakow, Poland; piotr.bomba@inbiosens.pl; 4 Polish American Heart of Poland, 47-200 Kedzierzyn-Kozle, Poland; stachurajarek@gmail.com; 5 Division of Cardiology and Structural Heart Diseases, Medical University of Silesia, 40-635 Katowice, Poland; p.m.gasior@gmail.com (P.G.); harpula.jan@gmail.com (J.H.); 6 Cardiology Department, George Papanikolaou General Hospital, 570 10 Thessaloniki, Greece; zarifis.john@gmail.com; 7 Department of Gastroenterology, Pomeranian Medical University, 71-455 Szczecin, Poland; marlicz@hotmail.com; 8 Department of Cardiac Surgery, Upper-Silesian Heart Center, 40-635 Katowice, Poland; damhud@gmail.com; 9 Interventional Cardiac Electrophysiology Group, International Clinical Research Center, St. Anne’s University Hospital Brno, 602 00 Brno, Czech Republic

**Keywords:** robotics, cardiology, interventional cardiology, electrophysiology, CABG, congenital heart disease

## Abstract

Robots are defined as programmable machines that can perform specified tasks. Medical robots are emerging solutions in the field of cardiology leveraging recent technological innovations of control systems, sensors, actuators, and imaging modalities. Robotic platforms are successfully applied for percutaneous coronary intervention, invasive cardiac electrophysiology procedures as well as surgical operations including minimally invasive aortic and mitral valve repair, coronary artery bypass procedures, and structural heart diseases. Furthermore, machines are used as staff-assisting tools to support nurses with repetitive clinical duties i.e., food delivery. High precision and resolution allow for excellent maneuverability, enabling the performance of medical procedures in challenging anatomies that are difficult or impossible using conventional approaches. Moreover, robot-assisted techniques protect operators from occupational hazards, reducing exposure to ionizing radiation, and limiting risk of orthopedic injuries. Novel automatic systems provide advantages for patients, ensuring device stability with optimized utilization of fluoroscopy. The acceptance of robotic technology among healthcare providers as well as patients paves the way for widespread clinical application in the field of cardiovascular medicine. However, incorporation of robotic systems is associated with some disadvantages including high costs of installation and expensive disposable instrumentations, the need for large operating room space, and the necessity of dedicated training for operators due to the challenging learning curve of robotic-assisted interventional systems.

## 1. Introduction

In the fast-paced world of healthcare, technological advancements continue to reshape the landscape of medical practices. The cardiovascular field has witnessed remarkable progress, where robots have emerged as transformative tools in the diagnosis, treatment, and management of diseases. Machines address some of the most significant challenges in clinical medicine, minimizing invasiveness and reducing the recovery time associated with traditional surgical procedures. By offering minimally invasive alternatives, robotic interventions remove the need for large incisions, resulting in reduced pain, decreased blood loss, and faster recovery times. This translates into shorter hospital stays, lower complication rates, and improved quality of life for patients.

Furthermore, the integration of telemedicine capabilities into robotic systems allows for remote patient monitoring and telesurgery, extending the reach of cardiovascular expertise beyond geographical boundaries. Patients in remote or underserved areas can now benefit from the expertise of highly skilled operators located elsewhere, thanks to the seamless collaboration facilitated by robots.

While the presence of robots in the field of cardiology and cardiac surgery is rapidly expanding, it is essential to address concerns surrounding the ethical, legal, and regulatory aspects of their use. Issues such as patient privacy, liability, and the proper training of healthcare professionals to operate these sophisticated machines require careful consideration to ensure their responsible integration into clinical practice.

As we delve deeper into this fascinating intersection of medicine and robotics, we can expect a future where robots play an increasingly vital role in safeguarding the health and well-being of millions worldwide. In this review, we outline the domains in which robotic technology is being used in cardiovascular care and analyze its benefits and drawbacks.

## 2. Coronary Interventional Cardiology

Since the first angioplasty treatment was carried out in 1977, percutaneous coronary intervention (PCI) has seen considerable advancements [1]. However, there have been no notable changes to the basic steps of the process used to execute PCI. Concerns about radiation exposure are raised by the complexity and increase in the number of coronary procedures, which necessitate close staff supervision. Lead aprons provide some partial protection for the operator from this exposure. These workplace dangers are to blame for both radiation-related illnesses (i.e., cataracts) and other non-radiation-related illnesses such as orthopedic injuries [2,3,4,5,6,7]. The “interventionalist’s disc disease”, which affects over half of all interventional operators, is a serious spine condition that could endanger patient treatment and career longevity [6,7].

The introduction of robotic PCI (R-PCI) (CorPath^®^ 200 Corindus, Siemens Healthineers Company, Waltham, MA, USA), addressed the risks to workers’ health connected with interventional cardiology [8,9]. The set-up included a radiation-shielded interventional cockpit inside the cardiac catheterization lab, together with monitors that showed hemodynamic data and fluoroscopic images. Cables link the robotic arm installed on the cardiac catheterization bedside rail to the interventional cockpit. A sterilized, one-time-use cassette makes up the robotic arm. The guiding catheter is advanced, and the coronary artery is engaged after the operator physically achieves access. The guide catheter is next connected to a robotic arm-mounted single-use cassette by the operator. Using the controls mounted on the cockpit console, the operator conducts the intervention while seated inside the interventional cockpit. These controls enable the operator to precisely maneuver guide catheters, balloons, and stents by applying longitudinal or rotational displacement to the control knobs [9].

R-PCI and manual percutaneous coronary intervention (M-PCI) were contrasted in the PRECISE (Percutaneous Robotically-Enhanced Coronary Intervention Study) trial [10]. In this one-center study, 80 consecutive patients who received M-PCI were compared to 40 patients who underwent R-PCI. All of the subjects had de novo obstructive coronary artery disease with angiographic stenosis greater than 50%, evidence of myocardial ischemia, and requirements for single-vessel PCI. Only two cases involving robotic assistance needed to be converted to manual PCI. The final residual stenosis for each patient was less than 30%. R-PCI was linked to trends toward reduced fluoroscopy time, radiation exposure, and contrast volume (Table 1).

Similar to the PRECISE experiment, the majority of treated lesions were short, non-type C lesions that could be treated with a single stent, raising concerns about the usefulness of R-PCI in complex coronary lesions. Due to this, 315 participants who underwent 334 PCI operations participated in the CORA-PCI Study (Complex Robotically Assisted Percutaneous Coronary Intervention) [11]. The 108 participants in the R-PCI had 157 lesions, of which 78.3% were B2/C lesions. A total of 226 individuals with 336 lesions, 68.8% of which were type B2/C, comprised M-PCI. R-PCI had a high (91.7%) and comparable clinical success rate to M-PCI. The rate of principal adverse events showed no discernible variations. Total contrast volume and patient radiation exposure were lower in the R-PCI group (Table 1). Furthermore, a noticeably longer process time was seen in the R-PCI. However, only procedures with minimal lesion complexity showed an increase in procedure time in the R-PCI group.

In comparison to manual PCI in patients with chronic total occlusion (CTO), Hirai et al. looked at the effects of robotic-assisted (RA) CTO PCI on procedure length and safety [12]. A total of 46 patients underwent an all-manual while 49 patients underwent RA CTO PCI. While there was no discernible difference in the use of contrast or the overall procedure time between the groups, the radiation dosage was lower in the RA CTO PCI group (Table 1).

Patel et al. compared R-PCI vs. M-PCI in a cohort of 996 consecutive patients in the largest trial to date [13]. A total of 310 patients (31.1%) underwent R-PCI, while 686 patients (68.9%) underwent conventional PCI. While the total procedure duration was significantly longer in the R-PCI group, the radiation dose was significantly lower. However, there was no distinction between the two groups in terms of fluoroscopy time or contrast volume.

In 2019, another R-PCI system R-One (Robocath, Rouen, France) received CE mark. The first human procedure was conducted in September 2019 in France. The results of the multicenter R-EVOLUTION (R-One Efficiency for PCI Evolution With Robotic Assistance) trial demonstrated safety and efficacy of the R-One robotic system for PCI in 64 patients with mostly non-complex (75%) de novo lesions with a significant reduction of radiation exposure to the operator [14]. No complications were reported during the procedure while successful R-PCI with no need to convert to M-PCI was achieved in 95% of the cases. Further studies are required to evaluate the safety and efficacy of the R-One system in a population with more complex lesions.

Finally, recent results of the first study evaluating an independently developed robot-assisted PCI system ETcath200 (WeMed Medical Equipment Co., Ltd., Beijing, China) were published [15]. Eleven patients with coronary heart disease referred for elective PCI were enrolled. The clinical success rate was defined as residual stenosis <30% after R-PCI, and no MACE up to 30 days following the procedure was 100%, with no PCI-related complications and no in-hospital or 30-day major adverse cardiovascular events. Furthermore, technical success defined as R-PCI without conversion to M-PCI was achieved in all patients. In summary, this study demonstrated the initial safety and feasibility of the ETcath200, however larger trials are required for this device to obtain regulatory approval in other markets.

## 3. Electrophysiology

Since the first electrophysiology (EP) procedure in 1967 [16], a very fast exploration of the basic electrophysiological concepts combined with incremental technological advances established radiofrequency ablation (RFA) a first-line therapy for simple and complex arrhythmias revolutionizing invasive treatment approaches. However, RFA procedures can be time-consuming and associated with high fluoroscopic exposure to both the patient and physician. It is clear that electrophysiologists face the same occupational hazards as the interventionists.

Over the past two decades, robotic systems have been built to ameliorate many of these potential complications substituting hand maneuvering of intracardiac catheters with machine-driven mapping and ablation technique. Four robotic electrophysiology solutions including remote magnetic navigation (RMN) systems (Stereotaxis Niobe/Genesis, St. Louis, MO, USA; CGCI, Inglewood, CA, USA), and manual robotic navigation (Sensei Robotic Navigation System, Mountain View, CA, USA; AMIGO Remote Catheter System, Fort Mill, SC, USA), among which Stereotaxis (St. Louis, MO, USA) is the most widely implemented and still developed EP robotic tool in clinical practice [17].

**Figure 1:**
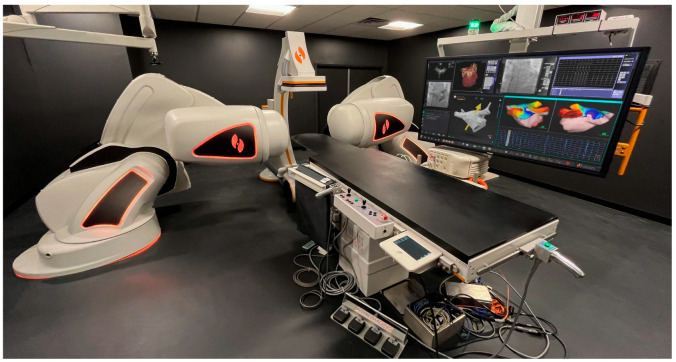
Genesis system, Stereotaxis, St. Louis, MO, USA [20].

The Genesis, newest version of the Stereotaxis RMN system introduced in 2020, is composed of two large magnets generating a uniform magnetic field of 0.08–0.1 T within the patient’s thorax (Figure 1). A steerable magnetic field gradient allows for three-dimensional (3D) navigation of magnetically compatible catheters. With a tremendous improvement in robotic technology, the third-generation quadripolar catheters with three magnets embedded in a distal tip (Celsius/Navistar RMT, Biosense Webster, Irvine, CA, USA; Magnoflush, Medfact, Germany; Trignum Flux, Biotronik, Berlin, Germany) enable omnidirectional maneuverability through reorientation of the external magnets and subsequent changes of magnetic vector with high (1 mm) spatial resolution when repositioning the catheter [18]. The operator can manipulate sheaths and diagnostic catheters by advancing or retracting the devices within cardiac chambers using a Vdrive Stereotaxis motor drive system, which ensures atraumatic catheter tip-tissue contact, excellent stability, and maneuverability translating into reduced catheter-induced ectopy and more durable lesions [19].

Importantly, in comparison to the previous version (Niobe, Stereotaxis), the Genesis system is 70–80% faster in manipulation of catheters through an intuitive computer interface located outside the operating room, providing almost instantaneous responsiveness controlling [21]. Furthermore, the Genesis system includes the integrated Model S fluoroscopy C-arm and Open Mapping API architecture, which is compatible with invasive 3D electro-anatomical mapping systems (CARTO, Biosense Webster, Diamond Bar, CA, USA; EnSite, Abbott, Abbott Park, IL, USA; Rhythmia, Boston Scientific, Marlborough, MA, USA) and the non-contact AcQMap mapping platform. Furthermore, it is synchronized with preoperative advanced EP planning tools (i.e., VIVO Catheter Precision, inHEART and ADAS 3D software) [22] and integrated with intracardiac echocardiography.

As mentioned before, RMN provides superior safety, catheter maneuverability, and significantly reduced fluoroscopy time in comparison to manual RFA procedures. Due to continuous technological improvement, current RMN systems offer a significant decrease in procedure time, with the recent studies indicating comparable duration of RMN vs. manual ablations [23,24]. Importantly, pivotal optimizations and substantial experience in the field of robotic EP have ensured high procedural effectiveness.

RMN is a viable alternative to traditional manual catheter ablation in patients with ventricular arrhythmias (VA). A recent meta-analysis comparing RMN and manual approach for ventricular tachycardia (VT) ablation (13 studies, over 1300 patients) showed higher acute ablation success rates, significantly lower rates of complications and reduced utilization of fluoroscopy in the RMN group [25]. Moreover, novel studies evaluating RMN vs. manual ablation in patients with VA reported a comparable total procedure time, which is crucial for EP lab logistics [26,27]. So far, manual percutaneous catheter ablations have been considered an effective strategy to treat premature ventricular contractions (PVC) [28]. However, the conventional approach for PVCs, in which the substrates originate from anatomically difficult locations, still remains a challenge. Apart from relatively approachable PVCs from the right ventricle and right ventricular outflow tract, RMN was found clinically useful for VA from the left ventricle (LV) [26,29,30,31,32]. In the study by Di Biase et al., RMN-driven ablations were performed antegradely and retrogradely, including substrates at aortomitral continuity, left coronary cusp, coronary sinus, and mitral valve (MV) annulus [33]. Studies applying RMN with open-irrigated-tip catheters for PVC/VT ablation show improved acute success rate ranging from 80 to 94% with comparable/outperforming results with the RMN approach in comparison to manual ablation in patients with PVCs [26,27,28]. Additionally, magnetic catheters have an advantage over manual catheters in case of epicardial access, as RMN-guided navigation provides better control over tip location in pericardial space where no torque points are available, limiting the useability of standard catheters [34].

**Figure 2:**
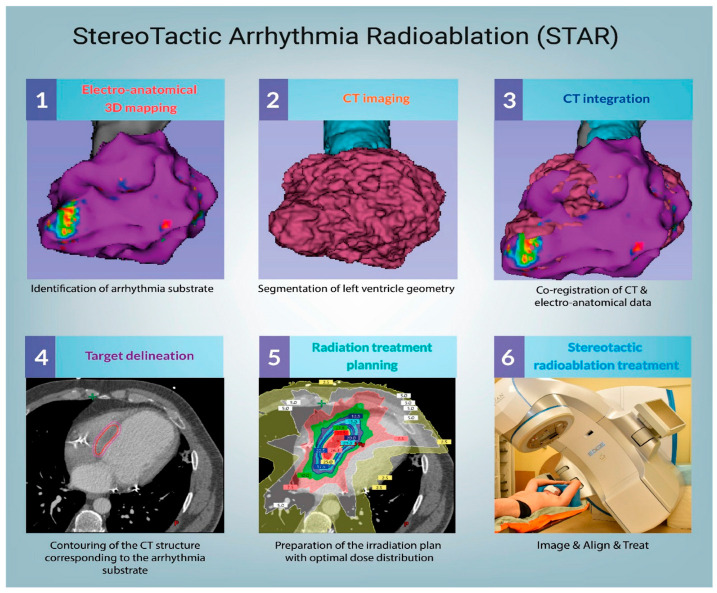
STAR treatment workflow. Reprinted from [35].

Apart from RMN systems, a novel treatment strategy has emerged for patients with VT refractory to anti-arrhythmic medications and conventional RFA. Experimental stereotactic arrhythmia radioablation (STAR) provides a non-invasive approach based on delivery of a large radiation dose to the region of arrhythmia substrate [35]. A single-fraction dose, usually 25 Gy, is considered ablative by triggering the acute radiation-mediated reprogramming of cardiac conduction (increased conduction velocity) [36] and long-term fibrotic response [37,38]. STAR exemplifies excellent multi-field collaboration between general cardiologists, electrophysiologists, radio-oncologists and interventional cardiologists while performing diagnostic evaluation, substrate localization and treatment planning. In most of the clinical trials, EP-guided cardiac irradiation procedures were performed using C-arm linear accelerators or CyberKinfe. Automatically, these systems deliver energy based on a pre-programmed treatment plan that targets specific anatomical locations utilizing respiratory compensation to reduce the impact on the surrounding organs. Figure 2 illustrates a workflow of STAR preparation and treatment. So far, published results indicate favorable clinical outcomes with acceptable risk [34]. The recent meta-analysis including 61 patients with intractable VT showed a 92% reduction of VT burden, and a 86% reduction in the number of implantable cardioverter-defibrillator shocks after STAR therapy. Moreover, the rate of grade 3 radioablation-associated adverse events was low, occurring in 2% of individuals with no grade 4–5 toxicity. Overall, the cardiac-specific survival at 6 months was 87%, which indicates promising treatment efficacy and preferable safety profile for a selected group of patients [39]. Despite satisfactory short-term outcomes, long-term follow-up results remain to be evaluated. Furthermore, to homogenize study protocols, a pan-European STOPSTORM project includes 31 centers to assess the clinical efficacy of the STAR treatment [40].

## 4. Cardiac Surgery

### 4.1. Robotic System for Cardiac Surgery Procedures

**Figure 3:**
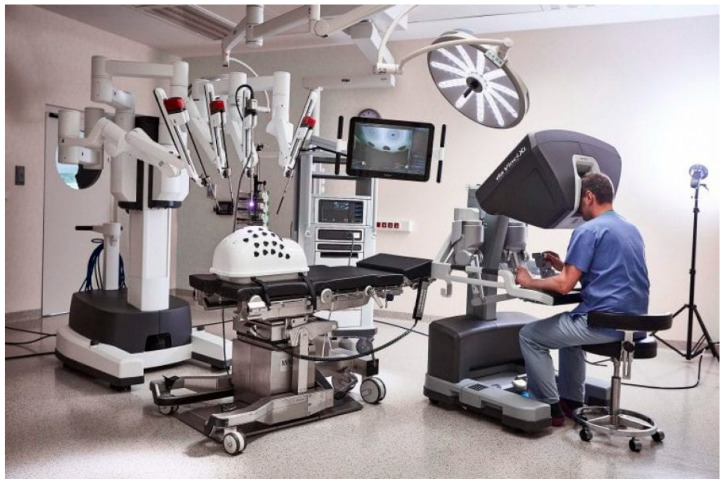
The da Vinci Surgical System, Intuitive Surgical, Sunnyvale, CA, USA [43].

The da Vinci Surgical System (Intuitive Surgical, Sunnyvale, CA, USA) is one of the most advanced robotic platforms used for cardiac surgery operations. This system is composed of a console (through which the surgeon performs the operation), a high-definition 3D vision system that can magnify the image up to 10 times, and a side robotic cart that is put by the patient’s side and has four robotic arms that the surgeon can control from a console (Figure 3). The surgical instruments are attached on three of the robotic arms, and the fourth robotic arm is utilized to operate the camera. The surgical instruments of the da Vinci^®^ system provide six degrees of freedom in comparison to traditional endoscopic surgery technologies, enabling complicated endoscopic surgical maneuvers such as sewing a vascular micro anastomosis. In laparoscopic or video-assisted thoracic surgery, the 3D endoscope provides greater depth awareness than the traditional 2D endoscopes. The operating field is essentially invisible to the surgeon at the console. Additionally, surgeons have independent control over the camera’s direction and positioning from the table-side surgical team. The second- and third-generation versions of these instruments are the da Vinci S^®^ and da Vinci Si^®^ systems, which compared to the first-generation model provide simpler arm movements and greater instrument reach. The company’s fourth generation model is the da Vinci Xi^®^ model. In this system, the added features include modification to the overhead architecture/patient-side cart with a boom mount so that multi-quadrant surgery can be performed without extensive repositioning, a laser targeting system to improve the ease of robotic arm set up, thinner arms/instruments with longer reach, and a movable endoscope which can be positioned on any of the four robotic arms [41,42]. Until now, in the field of cardiac surgery, there is no published study comparing the perioperative outcomes for procedures using the latest generation of the da Vinci robot versus its previous version.

### 4.2. Coronary Artery Bypass Graft Surgery

The effects of robotic techniques on coronary artery bypass graft (CABG) outcomes were evaluated by a retrospective examination of a nationwide database from US institutions [44]. The Nationwide Inpatient Sample yielded a weighted sample of 484,128 patients undergoing isolated coronary artery surgery, whereas 2582 of these patients underwent the procedure using robotic techniques (0.4%). A single bypass was performed in 59% of robotic cases, while two bypasses were performed in 25% of those cases. Patients who underwent robotic single bypass surgery compared to conventional surgery had lower postoperative stroke (0.0% vs. 1.5%, *p* = 0.045) and transfusion rates (13.5% vs. 24.4%, *p* = 0.001). Higher mortality (1.1% vs. 0.5%) and cardiovascular problems (12.2% vs. 10.6%) were noted in patients undergoing repeated bypass grafts when robotic assistance was used, although the changes were not statistically significant (*p* = 0.5).

Yokoyama et al. produced 7355 well-matched pairs of patients who underwent robotic and non-robotic CABG in the United States using the National Inpatient Sample [45]. According to the data, non-robotic CABG patients had a greater in-hospital death rate than robotic CABG patients (2.1% vs. 1.1%, *p* = 0.029). The rates of postoperative hemorrhage (25.9% vs. 20.3%, *p* = 0.044), acute kidney injury (15.8% vs. 12.3%, *p* = 0.0079), transfusion (24.3% vs. 11.0%, *p* = 0.0079), and duration of stay (9.3 ± 6.6 vs. 7.3 ± 6.2, *p* < 0.01) were also greater for non-robotic surgery. Additionally, individuals who had non-robotic surgery paid more overall (USD 46,196 vs. USD 41,317, *p* < 0.01).

In 281 patients who received robot-assisted CABG and 235 patients undergoing traditional CABG, Lin et al. looked at the long-term clinical results [46]. Following hospital release, the robotic-assisted CABG group and the traditional CABG group were followed for, respectively, 5.7 ± 3.0 and 5.0 ± 2.9 years on average, with statistical significance (*p* < 0.001). The in-hospital and long-term mortality rates were lower in the robot-assisted CABG group, but there was no appreciable difference between the two groups in the frequency of target lesion revascularization, target vessel revascularization, myocardial infarction, or stroke.

In a recent multicenter trial from the Netherlands, 194 patients who underwent traditional off-pump CABG were compared to 107 patients who underwent robot-assisted CABG [47]. All of the patients had single-vessel disease and had a surgery connecting the left internal mammary artery to the left anterior descending coronary artery. The robot-assisted group’s median postoperative hospital stay was 5 days as opposed to 7 days for the conventional off-pump group (*p* < 0.01). One month after the operation, the quality of life was comparable.

### 4.3. Mitral Valve Repair

Mihaljevic et al. show that robotically aided MV repair is just as secure and successful as repair carried out via full, partial, or mini-right thoracotomies [48]. The study group included 759 patients who underwent primary isolated MV repair at Cleveland Clinic using a robotic approach (*n* = 261), a mini-anterolateral thoracotomy (*n* = 114), a complete sternotomy (*n* = 114), or a partial sternotomy (*n* = 270). A robotic sternotomy took 42 min longer than a complete sternotomy, 39 min longer than a partial sternotomy, and 11 min longer than a right mini-anterolateral thoracotomy in matched groups (*p* < 0.0001). All matched groups’ MV repairs were of comparable quality. There were similarities between groups in terms of neurologic, pulmonary, and renal problems. The median hospital stay for the robotic group was 4.2 days, which was 1.0, 1.6, and 0.9 days shorter than for complete sternotomy, partial sternotomy, and right mini-anterolateral thoracotomy, respectively (all *p* < 0.01). The robotic group also had the lowest rates of atrial fibrillation and pleural effusion.

The clinical results and hospital costs of robotic vs. thoracoscopic mitral valve plasty (MVP) procedures were compared in a retrospective study from China [49]. In 121 patients, minimally invasive MVP was carried out using a robotic technology, and in 113 patients, it was carried out using a thoracoscopic technique. The thoracoscopic group experienced longer cardiopulmonary bypass (CPB) and aorta clamping times than the robotic group (153.2 ± 25.6 vs. 123.8 ± 34.9 min and 111.8 ± 23.0 vs. 84.9 ± 24.3 min, respectively; *p* < 0.001). In terms of total mortality, there was no discernible difference between the two groups. The thoracoscopic group had lower intraoperative blood transfusion rates (52.2% vs. 64.5%) and shorter intensive care unit stays (2.8 ± 2.3 vs. 3.6 ± 2.7 days, all *p* < 0.05) (2.8 ± 2.3 vs. 3.6 ± 2.7 days, all *p* < 0.05) than the robotic group. Last but not least, the robotic MVP group’s initial and adjusted operating room and hospital costs were significantly higher than those of the thoracoscopic MVP group (all *p* < 0.001).

Hawkins et al. evaluated the results of robotic, minimally invasive, and traditional mitral surgery by extracting cases undergoing non-emergent MV procedures from a local Society of Thoracic Surgeons database [50]. A total of 372 patients who underwent robotic mitral surgery compared favorably to 295 patients who underwent minimally invasive mitral surgery, 314 patients who underwent conventional MV surgery, and 314 patients who underwent robotic surgery. Mitral repair rates were high (91%) in the robotic and micro cohorts, but significantly lower (76%) in the traditional cohorts, *p* < 0.0001. The operative time was the longest of all the procedural procedures in the robotic cohort (224 vs. 168 min conventional, 222 vs. 180 min micro; all *p* < 0.0001). Last but not least, compared to traditional strategy, the postoperative duration of stay was shorter with robotic approach, but longer with the micro approach.

In a recent retrospective analysis, Barac et al. compared the outcomes of 129 patients who underwent robotic mitral repair vs. 628 patients who underwent port-access mitral repair for both in-hospital and intermediate-term outcomes [51]. According to a propensity score analysis of matched patients, those who underwent robotic mitral repair had longer pump and clamp times (275 ± 57 vs. 207 ± 55, *p* < 0.0001 and 152 ± 38 vs. 130 ± 34, respectively). However, there was no difference in the length of stay, postoperative morbidity, or 5-year survival (97 ± 1% vs. 96 ± 3%, *p* = 0.7) between the two groups. The incidence of severe mitral regurgitation (6 ± 4% vs. 1 ± 1%) and mitral reoperation (3 ± 2% vs. 1 ± 1%) over the course of 5 years was comparable among the matched individuals with degenerative valve disease. Finally, connective tissue illness, functional aetiology, and non-White race were predictors of recurrent moderate mitral regurgitation, but not surgical technique.

### 4.4. Robotic Aortic Valve Replacement

Currently, robotic aortic valve replacement (RAVR) is not as popular as robotic mitral valve surgery. This is due to the very good results of transcatheter aortic valve implantation (TAVI) procedures in high and medium risk patients and the increasing use of minimally invasive surgical approaches, such as partial sternotomy and right anterior thoracotomy (RAT) [52,53]. Some clinical situations limit the possibility of performing both TAVI and RAT. For TAVI procedures, these are bicuspid aortic valve (BAV) with large calcifications and the risk of paravalvular leakage, patient age <65 years, low operative risk, no calcification of the native aortic valve, horizontal position of the aorta, dilatation of the ascending aorta, stenosis in the left ventricle outflow tract, and the need for additional cardiac surgery procedures. Severe lung diseases such as emphysema or chronic obstructive pulmonary disease and frailty syndrome may be contraindications to minimally invasive aortic valve replacement (mini-sternotomy, RAT). The first step towards the robotization of aortic valve surgery was made by Folliguet, who in 2004 successfully performed a robot-assisted aortic valve replacement procedure in 5 patients. The procedures were performed via an anterior right mini--thoracotomy in the second intercostal space using the Da Vinci Surgical system (Intuitive Surgical, Inc., Sunnyvale, CA, USA) [54,55]. The first totally endoscopic robotic aortic valve replacement in humans was described in 2020 by Balkhy. The sutureless Perceval aortic valve prosthesis (Liva Nova, Saluggia, Italy) was implanted in a 76-year-old man with significant symptomatic aortic valve disease. The procedure was performed by right anterior mini-thoracotomy access using the Da Vinci Si^®^ surgical robot platform (Intuitive Surgical Inc., Sunnyvale, CA, USA) [56]. In 2022, Badwhar et al. published the first series of 50 patients who underwent aortic valve surgery using the fully RAVR technique [57]. Based on their experience with robotic mitral surgery, they used the right lateral thoracotomy (4th intercostal space) access and Da Vinci Xi^®^ robot platform (Intuitive Surgical Inc, Sunnyvale, CA, USA) with 4 ports (1 camera port and 3 working ports). It seems that the approach to RAVR, based on the experience with the platform for robotic mitral surgery, is the most interesting option at the moment. It is supported by good knowledge and excellent mastery of the robot system and the possibility of using various types of valve prostheses [57]. Peripheral cannulation for CPB in RAVR procedures, both arterial (femoral artery) and venous (bicaval: right femoral vein and right jugular vein) are commonly used in many cardiac centers for other procedures such as minimally invasive mitral valve surgery or extracorporeal membrane oxygenation therapy. Other technical aspects, such as the method of intubation (single or double lumen tube), the type and method of cardioplegia administration (antegrade or retrograde), and the type of aortic clamp used, depend on the patient’s anatomy and the individual experience of the centre. RAVR seems to be an interesting alternative for patients who are not good candidates for sternotomy. However, it is important to remember about potential problems and limitations. According to Badwhar, patients with coronary artery disease requiring surgical revascularization, patients with prior cardiac surgery or right thoracotomy, patients with peripheral vascular disease precluding CPB cannulation, and patients with a left ventricular ejection fraction of less than 25% are not eligible for RAVR [57]. However, aortotomy closure, annular suture placement, valvectomy and aortotomy are potential technical difficulties [58]. In addition, Sun points to the lack of a proper hook to expose the valve, difficulties in suturing the aortic annulus and long knotting time, as well as difficult cooperation with the assistant. However, as he himself notes, this may be due to the team’s limited experience [59]. In reports on RAVR, the achieved aortic clamping time and CPB time were much longer than in classic surgical aortic valve replacement procedures. This has a direct impact on the duration of cardiac ischaemia and the procedure [57,58,59]. Therefore, RAVR is not a good choice for patients with contractility disorders of the heart, low left ventricular ejection fraction, and high operative risk. However, the benefit of no sternotomy, no complications in wound healing, short hospitalization, quick rehabilitation, and good cosmetic effect may be an interesting alternative for a selected group of patients. The advantage of RAVR over minimally invasive surgical aortic valve replacement may be the enlarged 3D operating field, the use of a mechanical arm with 7 degrees of freedom, the reduction of incision size, and the elimination of hand tremor, which seems to be less important in aortic valve surgery than in coronary surgery [59]. In our opinion, in order to standardize and disseminate the method, it is necessary to create multi-center large registries of patients with aortic valve disease treated with RAVR.

## 5. Intracardiac Shunts

Atrial septal defect (ASD) is one of the commonest congenital heart defects [60]. Transcatheter ASD closure, if feasible, remains the standard of care because of its less invasive character. The size, position, and anatomy of some ASDs may preclude the use of a transcatheter approach and thus surgical repair is needed [61]. Conventional surgical methods with sternotomy or minimal thoracotomy are the common practice in such cases. However, several centers are now building up their experience in minimally invasive robot-assisted surgical techniques for the repair of some ASD cases [62]. Such techniques have been more commonly used for other conditions where they were shown to have a series of advantages over the conventional surgical methods [63,64,65]. Such advantages include smaller incisions, shorter recovery times, greater patient satisfaction, and better cosmetic outcomes [65,66,67,68].

Initially, the use of robotic surgical techniques was limited in the repair of secundum type ASD, whereas patients with more complicated types of atrial defects accompanied by partial anomalous pulmonary venous return (PAPVR) were not considered to be suitable candidates [69]. However, there are now reports of the feasibility and efficiency of robotic surgical repair of ASD with PAPVR [70]. The single-patch and the double-patch repair techniques have both been used depending on the type of defect. The surgical results were shown to be excellent and minimal risk for complications such as phrenic nerve injury, embolic events, superior vena cava obstruction or rhythm disturbances has been reported [71].

Under non-beating heart conditions, the robot-assisted ASD repair entails some challenges when compared to the traditional surgical approaches. For example, aortic cross-clamping through a small incision and root vent catheter use for cardioplegia infusion and deairing are more difficult than in the sternotomy method [72,73,74]. To overcome such difficulties, robot-assisted ASD repair with the heart beating has been advocated [75,76]. The question in the heart-beating approach is the potential risk of cerebral air embolism. Recently, Yun et al. have reported that robot-assisted ASD repair under heart beating conditions can be safely performed without additional risks [75]. They compared a group of 27 patients who underwent robotic ASD repair with the heart beating with a group of 18 patients in whom the robot-assisted repair was performed under non-heart beating conditions. There was no need for thoracotomy or median sternotomy conversion in any of the cases in the two groups and no residual ASD was detected postoperatively. Moreover, no additional risk for stroke due to air embolisms was shown in the beating heart patients in whom the CPB time was also shorter. However, the small number of patients in this cohort is a limitation and the safety and efficiency method will need to be assessed in larger groups of patients.

Despite the growing evidence of the feasibility, efficiency, and safety of a robot-assist surgical approach in the repair of ASD and the anticipated potential advantages over the conventional surgical methods, no up-to-date studies exist which directly compare the outcomes of the two approaches. A comparative study by Zheng et al. reports a multi-center experience from the period between September 2010 and June 2012 [77]. In this study, 254 patients with isolated ASD who underwent robot-assisted thoracoscopic surgical approach were compared with 254 patients who followed an open ASD surgical repair with median sternotomy. The robotic surgical approach group required longer aortic clamp time but had shorter durations of mechanical ventilation, intensive care unit stay and hospitalization. The CPB time was not shown to be significantly different in the two groups. The early outcomes for isolated ASD repair were similar for the two approaches while there were also no significant differences in mortality and in major in-hospital complications. However, since the publication of this study a lot more experience has been gained in the robot-assisted surgical ASD repair and novelty methods have been employed to address several issues.

Further multicenter comparative studies that include the most recent and continuous growing experience in the field of robotic surgical ASD repair are needed. Such studies should focus on comparing the robotic with the conventional surgical methods of repair—not only of isolated ASDs but also of more complicated ASD types that are associated with other heart defects such partial PAPVR or MV abnormalities. The aim will be to assess the potential advantages of one approach over the other in relation to clinical outcomes, safety, patient satisfaction, and cost-effectiveness, taking into consideration the effect of learning curve needs, the degree of robotic surgical systems availability to heart centers as well as potential procedural challenges and newly developed strategies to overcome them [78,79]. Such comparative studies will also help develop optimal patient selection criteria.

As previously mentioned, the standard preferred practice in ASD repair is the transcatheter approach, if this is feasible. The benefits of a transcatheter over surgical ASD closure include lower morbidity and mortality rates, shorter hospitalization duration, avoidance of CPB, and less cosmetic issues [80,81,82,83]. However, a transcatheter approach cannot be applied in cases of large secundum ASDs, of ASDs with deficient rims, or in other types of ASDs which are often also associated with pulmonary venous or MV abnormalities [61]. As robot-assisted ASD surgical repair has been increasingly applied as a least invasive, efficient, and safe alternative to conventional surgical sternotomy or minimal thoracotomy approaches and has been shown to be associated with increased patient satisfaction, the arising question is how it performs in comparison to the transcatheter approach.

Kadirogullari et al., in their retrospective analysis of a single-center experience, compared the outcomes of 217 patients who underwent robotic endoscopic ASD closure with those of 245 transcatheter ASD closure cases [84]. Both methods had good clinical outcomes with no mortality. The transcatheter approach group had a shorter duration of intensive care unit stay and hospitalization. The risk of post-procedural complications including neurological events, rhythm disturbances, and need for re-intervention was low and similar for the two approaches. Therefore, robot-assisted ASD repair has comparable outcomes with the transcatheter approach, which additionally entails the risk of complications such as device embolization or malposition, chronic erosion, and migraine due to nickel allergy [44,45,46]. Of course, more experience is needed before conclusions are derived over the clinical, cost-effectiveness, and accessibility advantages of one method over the other. Considering the advantages in patient comfort and satisfaction of the robotic approach over the conventional surgical techniques, robot-assisted ASD closure may be an efficient and safe alternative for those cases in which ASD size, location, and anatomy precludes a transcatheter approach.

Although significant experience has been gained so far in robot-assisted ASD closure in several centers, reports on robotic ventricular septal defect (VSD) repair are limited. The study with the largest population is that of Gao et al. which was published in 2012 [85]. In this study, 20 adult patients underwent successful endoscopic robot-assisted VSD surgery and there was no need in any of the cases to convert to open surgery and there were no residual ventricular septal defects postoperatively. Complications such as tricuspid valve regurgitation and atrioventricular conduction block were not seen in this cohort. Moreover, all patients went back to normal function within 1 week after the procedure.

Noteworthy, current robotic technology for ASD and VSD repair procedures is available for adult patients and not suited for the pediatric population for which specific surgical robots need to be developed.

## 6. Other Cardio-Thoracic Procedures

Besides the most common cardiac surgeries described above, there are procedures that also adopt a robotic approach. For such, the left atrium myxoma resection, as its characteristics requires flexible equipment and endoscopic technique, the comparative study performed by Schilling et al. showed that the successful procedures were comparable, yet the robotic assisted surgeries were linked to both shorter ICU (30.9 vs. 47.7 h) and hospital (3.6 vs. 6.2 days) stay [86]. The minimally invasive Cryo-Maze surgical treatment of atrial fibrillation, described firstly by Rodriguez et al., with equipment that could utilize robotic assistance in the future [87].

## 7. Other Robotic Surgical Devices

Undoubtedly the most widespread medical robot is the da Vinci system (described in Section 4.1), which has been on the market and in clinical practice for over 20 years at this point. However, as the medical robotic market is still developing, there are other manufacturers that produce their own solutions or engage in pre-clinical and clinical trials. Besides the devices described earlier in this review, other solutions in the robotic field are the Hugo RAS™ system (Medtronic, MN, USA) [88], approved by the FDA and EMA for laparoscopic surgeries and Johnson & Johnson with the Ottava™ [89], which is still in development. Apart from the biggest corporations, there are smaller manufacturers such as CMR Surgical (Cambridge, UK) with Versius robot [90] and Stryker (Kalamazoo, MI, USA) MAKO SmartRobotics designed for orthopedic procedures [91], Medicaroid (Kobe, Japan) offering hinotori™ Surgical Robot System for gastroenterology and gynecology applications [92], Titan Medical (Toronto, ON, Canada) with Enos 2.0 system, which would be suited for lower operating costs [93], the Moon Surgical (San Carlos, CA, USA) Maestro system [94] and Virtual Incision (Lincoln, NE, USA) MIRA platform [95], which ultimately will be placed on the International Space Station [96]. However, in the vast majority, the surgical robotic systems are firstly designed for urologic and gynecologic procedures, as they stand for over 50% of all surgeries performed with them. Designed for the cardiothoracic procedures, there is a RobInHeart platform, which is still in the development stage—however, as a part of presentation, an artificial teleoperation was performed with use of this device, with 13 km of distance between the operator and the robotic platform (with approximately 280 ms of vision latency) [97].

## 8. On-Ward Clinical Applications

Since the COVID-19 pandemic, many solutions have been invented or repurposed to improve the status of overwhelmed medical staff. This includes the various staff-assisting robots that were reviewed in this chapter. It includes mostly humanoid-shaped moving service robots. The focus of this chapter is the comparison of a few available robots (Table 2), the technologies they use (Table 3), and the barriers they had to overcome. We advise however to check each robot’s detailed descriptions and updates since they are actively developed in most of the cases. The methodology for the tables’ creation includes a review work by the author and assembling data published in articles and manufacturers’ specifications.

### 8.1. Common Tasks Solved by Staff-Assisting Robots

Staff-assisting robots can perform various tasks, but as shown in Table 2 (column 3) they are often specialized. In the realm of on-ward hospital robotics, a spectrum of operational functions collaborates to enhance patient care and facility throughput, operation and management. Alongside these core duties, auxiliary roles play a vital part in autonomous functioning, navigation and optimizing robot performance within the hospital environment. They are listed below:

1. Operational functions
  - Cleaning;
  - Data gathering—i.e., counting the number of people, screening tests;
  - Disinfection;
  - Information spreading;
  - Patrolling;
  - Reminding;
  - Social interaction—i.e., playing with children;
  - Telepresence;
  - Transport—i.e., drugs, food, other equipment, robotic arm movement, moving patients between beds;
  - Vitals monitoring.
2. Auxiliary functions
  - Simultaneous localization and mapping—required for all moving robots to navigate and move;
  - Multi-robot path planning;
  - Multi-robot task allocation;
  - Patient identification—required for delivery robots;
  - Movement;
  - Solving inverse kinematic problems—i.e., arm movement into desired location;
  - Own diagnostics.

**Figure 4:**
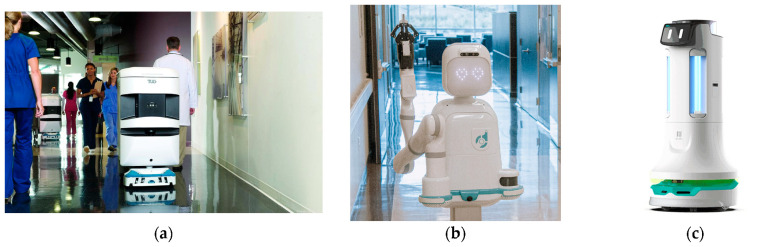
(**a**) Aethon TUG autonomous mobile robot (Aethon, Pittsburgh, PA, USA) [98]; (**b**) Moxi robot system (Diligent Robotics, Austin, TX, USA) [99]; (**c**) Pudu Puductor 2 disinfection robot (Pudu Robotics, Shenzhen, China) [100].

To exemplify, Figure 4 illustrates on-ward robotic systems used in practice.

In some cases, movement functions do not mean that the robot is a moving platform. A great example for this kind would be all robots that help while eating or feeding patients. My Spoon (SECOM Co., Tokyo, Japan) [101] and CareMeal (NT Robot Co., Seoul, Korea) [102] are stationary robots that move spoons attached to a robotic arm, while Liftware Level (Lift Labs of Verily Life Sciences LLC, San Francisco, CA, USA) is a robotic spoon that is a hand-held device [103].

Moreover, taking blood samples is one of the most frequently performed repetitive tasks in clinics. This is a relatively easy procedure with a low learning curve. However, it still requires a skilled medical professional with practical experience, especially for patients with unfavorable peripheral veins anatomy. A medical company Vitestro (Utrecht, The Netherlands) manufactured an autonomous blood drawing device that combines both ultrasound guidance and robotics. To this point, according to the manufacturer, the prototype of the main device was tested on more than 1000 patients, taking over 1500 blood samples [104]. However, there is no head-to-head comparison with manual blood drawing. The main objective, besides overcoming personnel shortages, is standardization of the process, as estimated costs of diagnostics errors could rise to over USD 160 million per year [104]. Furthermore, Leipheimer et al. described autonomous venipuncture system, a handheld device which was able to perform blood drawing with 87% success rate on all 31 participants, 97% in non-difficult venous access (25 patients), with a mean time of 93 s [105].

### 8.2. Challenges to Implement Nurse-Assisting Robots

Nurse-assisting robots have had to overcome certain barriers—one of them being technology. Since 2001, consumers have been able to purchase a Roomba cleaning robot [106], which is a good example of a service robot. However, the technology at the time was not yet ready for application in a medical environment. One of the most important challenges for the implementation of robots into a clinical space is associated with the readiness of healthcare workers for new technology. Specifically, for nurse-assisting robots it was reported that about 65.8% workers were positive about robots promoting delivery tasks [107]. The authors of the same article mentioned that not many robots were implemented during the COVID-19 pandemic, and suggest that they may not be the solution due to the lack of adaptability and changing user trends.

A recent report supports this argument. As of April 2022, a range of Aethon (Pittsburgh, PA, USA) hospital robots have been patched since several significant zero-day vulnerabilities (CVSS > 7.6 up to 9.8) were found [108]. They made it possible for hackers to obtain full control over robots and thus to take photos, gain access to the records, and block drug delivery tasks.

**Table 2:** Comparison of nurse assisting robots.

Name	Type	Specialization	Known Used Technology	Comment
Aethon TUG Door (T2) [109]	Moving	Delivery	3, 4, 7, 16, 17, 19, 20	T3 version is a newer platform, T4 version is as of writing the latest
Aethon TUG Drawer (T2) [109]	Moving	Delivery	3, 4, 7, 16, 17, 19, 20	Same platform as Aethon Door, different configuration
Aethon TUG T3/T3XL [110]	Moving	Delivery	1, 4, 7, 11, 12, 16, 17, 18, 19, 20	Actively developed, latest platform for medical purposes
Digilent Moxi [99]	Moving	Multi-purpose	11, 13, 16	Actively developed, no datasheet found. Website does not contain much technical information
Dinsaw [111]	Stationary	Elderly care	16, 20	Website not available in English, does not contain much technical information
Giraff [112]	Moving	Telepresence	-	No longer available for purchase
Grace [113]	Moving	Social	-	Mentioned in news, but not on the manufacturer’s website
Liftware Level [103]	Hand-held	Spoon	-	Sold out
Paro [114]	Stationary	Elderly care	-	Over 112 sold in US. Recent articles mentioning it were published in 2022
Pepper [115]	Moving	Multi-purpose	2, 4, 6, 7, 10, 13, 14, 16, 17	Datasheet available, compared to all other robots contains the highest amount of information
Pudu PuduBot 2 [100]	Moving	Delivery	1, 9, 11, 16, 20	In this case 900 MHz refers to LoRa standard of communication and not a GSM band
Pudu Puductor 2 [100]	Moving	Disinfection	-	Uses millimeter wave motion sensors, contrary to any other of the list

**Table 3:** Common technologies used in nurse-assisting robots.

No.	Technology	Description and Purpose
1	900 MHz	Frequency of communications used by some robots. Exact protocol and technology are not specified under this name because the frequency is internationally allocated to amateur radio and for 2G GSM voice and basic data communication. May represent LoRa 915 MHz. The name is taken as it was used in the Aethon T2 datasheet
2	Android operating system	Main operating system in many telepresence robots
3	Biometric access	Some delivery robots can be opened only with biometric identification. This includes fingerprint recognition
4	Cloud-hosted control panel	Robots are managed with a cloud-hosted web service, requiring internet connection. Data may be stored outside the hospital
5	Gyroscope	Stabilization of cameras, spoons, and telepresence screens
6	IMU	Inertial measurement unit for position and orientation tracking
7	Infrared proximity sensor	Used for the detection of objects (doors, walls)
8	Infrared reflectance sensor	Used for line followers to detect a line
9	Li-FePO4	Battery technology—has smaller energy density than Li-Ion, but it is much less prone to failure, much safer, and has a longer lifespan
10	Li-Ion	Battery technology, highest energy density
11	LIDAR	Used for detection of objects (doors, walls, people)
12	Locally hosted control panel	The robot management panel is hosted locally on the robot itself or on a server inside the hospital. No data should be sent outside. Less support may be provided by the manufacturer
13	Robotic arm	Used for feeding patients, drug delivery, small item handling
14	Stereoscopic camera	Used for detection or recognition of objects (doors, walls, people)
15	Thermal camera	Used for temperature measurements
16	Touchscreen	Touchscreen to manage the robot or communicate with patients. Multiple types and technologies are used
17	Ultrasonic proximity sensors	Used for the detection of objects (which includes transparent doors)
18	Valve-regulated Lead-Acid battery	Older technology batteries used as a main source of energy. Mostly obsolete to Li-Ion and Li-FePO4 due to low energy density
19	VPN connection	A secure, encrypted connection to remote servers
20	Wi-Fi	Communications technology

## 9. Discussion

The implementation of robotic solutions in the field of cardiovascular medicine holds a great promise. However, despite the potential impact on the healthcare sector, active robotic installations are available in a limited number of centers worldwide. This situation is associated with a trade-off between benefits (reduced radiation, applicability for complex cases) and limitations (high costs, learning curve-associated increased procedure time).

For robot-assisted PCI procedures, the operator’s exposure to radiation during procedures is significantly reduced, which is a big advantage for interventionists or electrophysiologists. Of all healthcare professionals, interventional cardiologists are exposed to the most radiation (up to 5 mSv annually, or 2–3 times more than interventional radiologists) [116,117]. Concerning instances of left-sided brain tumors in interventional cardiologists also raise the possibility of a link to radiation exposure over the course of a career [118]. Ionizing radiation also raises the incidence of cataracts, with one study finding that interventional cardiologists were 50% more likely to have posterior subcapsular alterations than age-matched controls with less than 10% [119]. Robotically assisted operations for interventionists or electrophysiologists greatly limit operator exposure to dangerous ionizing radiation without degrading the effectiveness of the procedure or the patient’s outcome. Robotics also offers the obvious advantage of reducing the risk of orthopedic injuries experienced by operators. By enabling more accurate lesion length measurement, precise stent placement, and less patient radiation exposure, robotically assisted interventions have the potential to benefit patients on a personal level.

Additionally, R-PCI offers technical advantages that may lessen the likelihood of longitudinal geographic miss or the failure to completely cover a sick coronary segment during PCI operations, both of which have a detrimental effect on the long-term clinical results of patients. Lesion length measurements are possible with the R-PCI system, using either a balloon catheter or, more frequently, a coronary angioplasty guidewire. Visual estimation of lesion length and stent length selection was 65% more erroneous than the robot-assisted platform [120]. Comparing patients undergoing R-PCI (PRECISE trial) with a patient group who had M-PCI, the incidence of longitudinal geographic miss was significantly lower (12.2% vs. 43.1%, respectively; *p* < 0.0001) [121].

Importantly, the capability of the robotic operator system to be operated remotely, or “tele-stenting”, is one creative application of R-PCI. When Madder and colleagues successfully performed R-PCI on 20 patients while a physician operator was stationed in a separate procedure room from the catheterization laboratory, they demonstrated that R-PCI tele-stenting was technically possible [122].

The first reports of robotic telemanipulation being used for CABG date back to the late 1990s. However, robotic coronary surgery has not gained much traction after an early rush of interest. Remarkably, it is now more feasible to conduct robotic surgery. Especially with tele-robotics, doctors can treat individuals from afar without compromising the quality of their services or the support they extend to their patients.

As an important advantage, robotic cardiac surgery has been proven to be safe and successful, with some treatments showing shorter hospital stays and decreases in complications when compared to conventional approaches. Robotic surgery is less invasive from the patient’s perspective, which results in less discomfort and a speedier recovery.

Robotic surgery reduces operator fatigue and reduces human mistakes by not being as taxing as traditional surgery. Cardiac operations can last for several hours and are taxing on the surgeon performing them. The surgeon may operate while sitting comfortably thanks to surgery robots. However, since robots are not human beings, they will not get fatigued. Their “hands” are frequently firm and unshakeable and will not tremble. They are able to operate on the patient for an extended amount of time without suffering any consequences regarding their stability, precision, or judgment. An obvious benefit of these systems and techniques is the high precision and accuracy of robotically aided, catheter-based, or surgical operations. However, it is still unclear whether this development would result in a better clinical outcome.

The learning curve of robotic techniques in cardiology is one of their key drawbacks. In electrophysiology, it is generally agreed that 20–50 examples are required to get through the initial learning curve. On the other side, surgeons frequently observe that mastering the da Vinci System has a much steeper learning curve and requires time (as described in the Section 4.1). Before the surgeon admits they have mastered the robot, it typically takes at least hundreds of operations [123,124]. Some people think that using this extra training time to hone a surgeon’s manual dexterity could be a better use of it.

The price of these systems is another drawback. Because it costs more to buy and set up surgical robots, robotic procedures are more expensive than traditional surgeries. It is unclear if the cost of these systems will go up or down in the future. Some people think that the price will decrease as technology advances and as more people get experience using robotic systems. Others think that the cost of these systems will go up as technology advances and more sophisticated software is introduced. Surgical robots have a fixed price and an annual maintenance fee, but if the robots are used frequently, the cost to patients will be significantly lower. Concentrating the application of the technology in specialized and, preferably, large volume centers, is a fair strategy. The significant costs associated with robotic devices can perhaps only be controlled through this concentration process. The question of how much money hospitals and other healthcare facilities will need to spend on system improvements and how frequently is another drawback. Technology is constantly evolving, and each year, new gadgets emerge that are better than their predecessors in terms of quality, enhancing the user experience. The medical equipment may also soon become outdated, necessitating a continuous replacement of the apparatuses and significantly raising the cost.

The issue of latency, which is the time lag between the surgeon’s instruction transmitted through the console and the robot accepting and carrying out the command by moving its arms as commanded, is another key concern with robotic surgery. The robotic arms and computer do not start talking to one another right away.

The benefits can eventually outweigh the drawbacks with the appropriate knowledge and tools. The cost of these devices is still considerable, and they do not make common treatments less expensive. However, even if the cost of the technique slows down its integration in hospitals, robotic surgery will continue to become more prevalent, allowing for more accurate and precise microsurgeries.

## Figures and Tables

**Table 1:** Procedural characteristics of robotic-assisted and totally manual PCI.

Study	Procedure (min)	Time	*p*-Value	Fluoroscopy (min)	Time	*p*-Value	Radiation		*p*-Value	Contrast (mL)	Volume	*p*-Value
	R-PCI	M-PCI		R-PCI	M-PCI		R-PCI	M-PCI		R-PCI	M-PCI	
Smilowitz 2014 [10]	n/a	n/a		10.1 ± 4.7	12.3 ± 7.6	0.05	1389 ± 599 mGy	1665 ± 1026 mGy	0.07	121 ± 47	137 ± 62	0.11
Mahmud 2017 [11]	44.3 ± 26	36.34 ± 23	0.002	18.2 ± 10.4	19.2 ± 11.4	0.39	12,518 ± 15,970 cGycm^2^	14,048 ± 18,437 cGycm^2^	0.045	183.4 ± 78.7	202.5 ± 74	0.031
Hirai 2019 [12]	89.6 ± 27.1	93.4 ± 30.5	0.52	37.9 ± 17.9	48.6 ± 17.1	<0.01	1522 ± 1129 mGy	2466 ± 1204 mGy	<0.01	111 ± 39	118 ± 53	0.47
Patel 2020 [13]	31 (21–42)	36 (26–49)	<0.0005	6.29 (4.23–9.68)	5.39 (3.17–9.03)	<0.0005	6313 (4049–9574) cGycm^2^	4465 (2644–7389) cGycm^2^	<0.0005	150 (120–190)	130 (100–170)	<0.0005

n/a—not available.

## Data Availability

No new dataset available for this review article.

## Abbreviations

2D	two-dimensional
3D	three-dimensional
ASD	atrial septal defect
BAV	bicuspid aortic valve
CABG	coronary artery bypass grafting
CPB	cardiopulmonary bypass
CTO	chronic total occlusion
EP	electrophysiology
ICU	intensive care unit
M-PCI	manual percutaneous coronary intervention
MV	mitral valve
MVP	mitral valve plasty
PCI	percutaneous coronary intervention
PAPVR	partial anomalous pulmonary venous return
PVC	premature ventricular contractions
R-PCI	robotic percutaneous coronary intervention
RA	robotic assisted
RAT	right anterior thoracotomy
RAVR	robotic aortic valve replacement
RFA	radiofrequency ablation
RMN	remote magnetic navigation
STAR	stereotactic arrhythmia radio ablation
TAVI	transcatheter aortic valve implantation
VA	ventricular arrhythmias
VSD	ventricular septal defect
VT	ventricular tachycardia

## References

[1] Grüntzig A.R., Senning A., Siegenthaler W.E. (1979). Nonoperative dilatation of coronary-artery stenosis: Percutaneous transluminal coronary angioplasty. N. Engl. J. Med..

[2] Roguin A., Goldstein J., Bar O., Goldstein J.A. (2013). Brain and neck tumors among physicians performing interventional procedures. Am. J. Cardiol..

[3] Ciraj-Bjelac O., Rehani M.M., Sim K.H., Liew H.B., Vano E., Kleiman N.J. (2010). Risk for radiation-induced cataract for staff in interventional cardiology: Is there reason for concern?. Catheter. Cardiovasc. Interv..

[4] Vano E., Kleiman N.J., Duran A., Rehani M.M., Echeverri D., Cabrera M. (2010). Radiation cataract risk in interventional cardiology personnel. Radiat. Res..

[5] Andreassi M.G., Piccaluga E., Gargani L., Sabatino L., Borghini A., Faita F., Bruno R.M., Padovani R., Guagliumi G., Picano E. (2015). Subclinical carotid atherosclerosis and early vascular aging from long-term low-dose ionizing radiation exposure: A genetic, telomere, and vascular ultrasound study in cardiac catheterization laboratory staff. JACC Cardiovasc. Interv..

[6] Goldstein J.A., Balter S., Cowley M., Hodgson J., Klein L.W. (2004). Occupational hazards of interventional cardiologists: Prevalence of orthopedic health problems in contemporary practice. Catheter. Cardiovasc. Interv..

[7] Klein L.W., Tra Y., Garratt K.N., Powell W., Lopez-Cruz G., Chambers C., Goldstein J.A. (2015). Occupational health hazards of interventional cardiologists in the current decade: Results of the 2014 SCAI membership survey. Catheter. Cardiovasc. Interv..

[8] Beyar R., Wenderow T., Lindner D., Kumar G., Shofti R. (2005). Concept, design and pre-clinical studies for remote control percutaneous coronary interventions. EuroIntervention J. EuroPCR Collab. Work. Group Interv. Cardiol. Eur. Soc. Cardiol..

[9] Beyar R., Gruberg L., Deleanu D., Roguin A., Almagor Y., Cohen S., Kumar G., Wenderow T. (2006). Remote-control percutaneous coronary interventions: Concept, validation, and first-in-humans pilot clinical trial. J. Am. Coll. Cardiol..

[10] Smilowitz N.R., Moses J.W., Sosa F.A., Lerman B., Qureshi Y., Dalton K.E., Privitera L.T., Canone-Weber D., Singh V., Leon M.B. (2014). Robotic-Enhanced PCI Compared to the Traditional Manual Approach. J. Invasive Cardiol..

[11] Mahmud E., Naghi J., Ang L., Harrison J., Behnamfar O., Pourdjabbar A., Reeves R., Patel M. (2017). Demonstration of the Safety and Feasibility of Robotically Assisted Percutaneous Coronary Intervention in Complex Coronary Lesions: Results of the CORA-PCI Study (Complex Robotically Assisted Percutaneous Coronary Intervention). JACC Cardiovasc. Interv..

[12] Hirai T., Kearney K., Kataruka A., Gosch K.L., Brandt H., Nicholson W.J., Lombardi W.L., Grantham J.A., Salisbury A.C. (2020). Initial report of safety and procedure duration of robotic-assisted chronic total occlusion coronary intervention. Catheter. Cardiovasc. Interv..

[13] Patel T.M., Shah S.C., Soni Y.Y., Radadiya R.C., Patel G.A., Tiwari P.O., Pancholy S.B. (2020). Comparison of Robotic Percutaneous Coronary Intervention With Traditional Percutaneous Coronary Intervention: A Propensity Score-Matched Analysis of a Large Cohort. Circ. Cardiovasc. Interv..

[14] Durand E., Sabatier R., Smits P.C., Verheye S., Pereira B., Fajadet J. (2023). Evaluation of the R-One robotic system for percutaneous coronary intervention: The R-EVOLUTION study. EuroIntervention J. EuroPCR Collab. Work. Group Interv. Cardiol. Eur. Soc. Cardiol..

[15] Zhai G.Y., Chen Z., Liu R.F., Guo Y.H., Wang J.L., Sun T.N., Xie J., Huang T., Zhou Y.J. (2022). First-in-human evaluation of an independently developed Chinese robot-assisted system for percutaneous coronary intervention. J. Geriatr. Cardiol. JGC.

[16] Furman S., Robinson G. (1958). The use of an intracardiac pacemaker in the correction of total heart block. Surg. Forum.

[17] Bassil G., Markowitz S.M., Liu C.F., Thomas G., Ip J.E., Lerman B.B., Cheung J.W. (2020). Robotics for catheter ablation of cardiac arrhythmias: Current technologies and practical approaches. J. Cardiovasc. Electrophysiol..

[18] Bhaskaran A., Barry M.A., Al Raisi S.I., Chik W., Nguyen D.T., Pouliopoulos J., Nalliah C., Hendricks R., Thomas S., McEwan A.L. (2015). Magnetic guidance versus manual control: Comparison of radiofrequency lesion dimensions and evaluation of the effect of heart wall motion in a myocardial phantom. J. Interv. Card. Electrophysiol..

[19] Pappone C., Vicedomini G., Frigoli E., Giannelli L., Ciaccio C., Baldi M., Zuffada F., Saviano M., Pappone A., Crisà S. (2011). Irrigated-tip magnetic catheter ablation of AF: A long-term prospective study in 130 patients. Heart Rhythm.

[20] Stereotaxis Genesis. https://www.stereotaxis.com/products/.

[21] Lin C., Pehrson S., Jacobsen P.K., Chen X. (2017). Initial experience of a novel mapping system combined with remote magnetic navigation in the catheter ablation of atrial fibrillation. J. Cardiovasc. Electrophysiol..

[22] Grace A., Willems S., Meyer C., Verma A., Heck P., Zhu M., Shi X., Chou D., Dang L., Scharf C. (2019). High-resolution noncontact charge-density mapping of endocardial activation. JCI Insight.

[23] Yuan S., Holmqvist F., Kongstad O., Jensen S.M., Wang L., Ljungström E., Hertervig E., Borgquist R. (2017). Long-term outcomes of the current remote magnetic catheter navigation technique for ablation of atrial fibrillation. Scand. Cardiovasc. J. SCJ.

[24] Maurer T., Sohns C., Deiss S., Rottner L., Wohlmuth P., Reißmann B., Heeger C.H., Lemes C., Riedl J., Santoro F. (2017). Significant reduction in procedure duration in remote magnetic-guided catheter ablation of atrial fibrillation using the third-generation magnetic navigation system. J. Interv. Card. Electrophysiol..

[25] Blandino A., Bianchi F., Sibona Masi A., Mazzanti A., D’Ascenzo F., Grossi S., Musumeci G. (2021). Outcomes of manual versus remote magnetic navigation for catheter ablation of ventricular tachycardia: A systematic review and updated meta-analysis. Pacing Clin. Electrophysiol. PACE.

[26] Shauer A., De Vries L.J., Akca F., Palazzolo J., Shurrab M., Lashevsky I., Tiong I., Singh S.M., Newman D., Szili-Torok T. (2018). Clinical research: Remote magnetic navigation vs. manually controlled catheter ablation of right ventricular outflow tract arrhythmias: A retrospective study. Eur. Eur. Pacing Arrhythm. Card. Electrophysiol. J. Work. Groups Card. Pacing Arrhythm. Card. Cell. Electrophysiol. Eur. Soc. Cardiol..

[27] Kawamura M., Scheinman M.M., Tseng Z.H., Lee B.K., Marcus G.M., Badhwar N. (2017). Comparison of remote magnetic navigation ablation and manual ablation of idiopathic ventricular arrhythmia after failed manual ablation. J. Interv. Card. Electrophysiol..

[28] Qiu X., Zhang N., Luo Q., Liu A., Ji Y., Ye J., Lin C., Ling T., Chen K., Pan W. (2018). Remote magnetic navigation facilitates the ablations of frequent ventricular premature complexes originating from the outflow tract and the valve annulus as compared to manual control navigation. Int. J. Cardiol..

[29] Cronin E.M., Bogun F.M., Maury P., Peichl P., Chen M., Namboodiri N., Aguinaga L., Leite L.R., Al-Khatib S.M., Anter E. (2020). 2019 HRS/EHRA/APHRS/LAHRS expert consensus statement on catheter ablation of ventricular arrhythmias. Heart Rhythm.

[30] Guckel D., Niemann S., Ditzhaus M., Molatta S., Bergau L., Fink T., Sciacca V., El Hamriti M., Imnadze G., Steinhauer P. (2021). Long-Term Efficacy and Impact on Mortality of Remote Magnetic Navigation Guided Catheter Ablation of Ventricular Arrhythmias. J. Clin. Med..

[31] Xie Y., Liu A., Jin Q., Zhang N., Jia K., Lin C., Ling T., Chen K., Pan W., Wu L. (2020). Novel strategy of remote magnetic navigation-guided ablation for ventricular arrhythmias from right ventricle outflow tract. Sci. Rep..

[32] Dang S., Jons C., Jacobsen P.K., Pehrson S., Chen X. (2019). Feasibility of a novel mapping system combined with remote magnetic navigation for catheter ablation of premature ventricular contractions. J. Arrhythmia.

[33] Di Biase L., Santangeli P., Astudillo V., Conti S., Mohanty P., Mohanty S., Sanchez J.E., Horton R., Thomas B., Burkhardt J.D. (2010). Endo-epicardial ablation of ventricular arrhythmias in the left ventricle with the Remote Magnetic Navigation System and the 3.5-mm open irrigated magnetic catheter: Results from a large single-center case-control series. Heart Rhythm.

[34] Burkhardt J.D., Di Biase L., Horton R., Schweikert R.A., Natale A. (2010). Remote Navigation and Electroanatomic Mapping in the Pericardial Space. Card. Electrophysiol. Clin..

[35] Miszczyk M., Jadczyk T., Gołba K., Wojakowski W., Wita K., Bednarek J., Blamek S. (2021). Clinical Evidence behind Stereotactic Radiotherapy for the Treatment of Ventricular Tachycardia (STAR)-A Comprehensive Review. J. Clin. Med..

[36] Zhang D.M., Navara R., Yin T., Szymanski J., Goldsztejn U., Kenkel C., Lang A., Mpoy C., Lipovsky C.E., Qiao Y. (2021). Cardiac radiotherapy induces electrical conduction reprogramming in the absence of transmural fibrosis. Nat. Commun..

[37] Refaat M.M., Ballout J.A., Zakka P., Hotait M., Al Feghali K.A., Gheida I.A., Saade C., Hourani M., Geara F., Tabbal M. (2017). Swine Atrioventricular Node Ablation Using Stereotactic Radiosurgery: Methods and In Vivo Feasibility Investigation for Catheter-Free Ablation of Cardiac Arrhythmias. J. Am. Heart Assoc..

[38] Lehmann H.I., Graeff C., Simoniello P., Constantinescu A., Takami M., Lugenbiel P., Richter D., Eichhorn A., Prall M., Kaderka R. (2016). Feasibility Study on Cardiac Arrhythmia Ablation Using High-Energy Heavy Ion Beams. Sci. Rep..

[39] Viani G.A., Gouveia A.G., Pavoni J.F., Louie A.V., Detsky J., Spratt D.E., Moraes F.Y. (2023). A Meta-analysis of the Efficacy and Safety of Stereotactic Arrhythmia Radioablation (STAR) in Patients with Refractory Ventricular Tachycardia. Clin. Oncol..

[40] A Prospective European Validation Cohort for Stereotactic Therapy of Re-Entrant Tachycardia. https://cordis.europa.eu/project/id/945119.

[41] Ferguson J.M., Pitt B., Kuntz A., Granna J., Kavoussi N.L., Nimmagadda N., Barth E.J., Herrell S.D., Webster R.J. (2020). Comparing the accuracy of the da Vinci Xi and da Vinci Si for image guidance and automation. Int. J. Med. Robot. Comput. Assist. Surg. MRCAS.

[42] Ojima T., Nakamura M., Hayata K., Kitadani J., Takeuchi A., Yamaue H. (2021). Comparison of short-term surgical outcomes using da Vinci S, Si and Xi Surgical System for robotic gastric cancer surgery. Sci. Rep..

[43] Healthcare Market Experts Robotics—The Future of Surgery. https://healthcaremarketexperts.com/en/news/joanna-szyman-for-pmr-robotics-the-future-of-surgery/.

[44] Cavallaro P., Rhee A.J., Chiang Y., Itagaki S., Seigerman M., Chikwe J. (2015). In-hospital mortality and morbidity after robotic coronary artery surgery. J. Cardiothorac. Vasc. Anesth..

[45] Yokoyama Y., Kuno T., Malik A., Briasoulis A. (2021). Outcomes of robotic coronary artery bypass versus nonrobotic coronary artery bypass. J. Card. Surg..

[46] Lin T.H., Wang C.W., Shen C.H., Chang K.H., Lai C.H., Liu T.J., Chen K.J., Chen Y.W., Lee W.L., Su C.S. (2021). Clinical outcomes of multivessel coronary artery disease patients revascularized by robot-assisted vs conventional standard coronary artery bypass graft surgeries in real-world practice. Medicine.

[47] Spanjersberg A., Hoek L., Ottervanger J.P., Nguyen T.Y., Kaplan E., Laurens R., Singh S. (2022). Early home discharge after robot-assisted coronary artery bypass grafting. Interact. Cardiovasc. Thorac. Surg..

[48] Mihaljevic T., Jarrett C.M., Gillinov A.M., Williams S.J., DeVilliers P.A., Stewart W.J., Svensson L.G., Sabik J.F., Blackstone E.H. (2011). Robotic repair of posterior mitral valve prolapse versus conventional approaches: Potential realized. J. Thorac. Cardiovasc. Surg..

[49] Wei S., Zhang X., Cui H., Zhang L., Gong Z., Li L., Ren T., Gao C., Jiang S. (2020). Comparison of clinical outcomes between robotic and thoracoscopic mitral valve repair. Cardiovasc. Diagn. Ther..

[50] Hawkins R.B., Mehaffey J.H., Mullen M.G., Nifong W.L., Chitwood W.R., Katz M.R., Quader M.A., Kiser A.C., Speir A.M., Ailawadi G. (2018). A propensity matched analysis of robotic, minimally invasive, and conventional mitral valve surgery. Heart.

[51] Barac Y.D., Loungani R.S., Sabulsky R., Zwischenberger B., Gaca J., Carr K., Glower D.D. (2021). Robotic versus port-access mitral repair: A propensity score analysis. J. Card. Surg..

[52] Smith C.R., Leon M.B., Mack M.J., Miller D.C., Moses J.W., Svensson L.G., Tuzcu E.M., Webb J.G., Fontana G.P., Makkar R.R. (2011). Transcatheter versus surgical aortic-valve replacement in high-risk patients. N. Engl. J. Med..

[53] Reardon M.J., Van Mieghem N.M., Popma J.J., Kleiman N.S., Søndergaard L., Mumtaz M., Adams D.H., Deeb G.M., Maini B., Gada H. (2017). Surgical or Transcatheter Aortic-Valve Replacement in Intermediate-Risk Patients. N. Engl. J. Med..

[54] Folliguet T.A., Vanhuyse F., Magnano D., Laborde F. (2004). Robotic aortic valve replacement: Case report. Heart Surg. Forum.

[55] Folliguet T.A., Vanhuyse F., Konstantinos Z., Laborde F. (2005). Early experience with robotic aortic valve replacement. Eur. J. Cardio Thorac. Surg..

[56] Balkhy H.H., Kitahara H. (2020). First Human Totally Endoscopic Robotic-Assisted Sutureless Aortic Valve Replacement. Ann. Thorac. Surg..

[57] Wei L.M., Cook C.C., Hayanga J.W.A., Rankin J.S., Mascio C.E., Badhwar V. (2022). Robotic Aortic Valve Replacement: First 50 Cases. Ann. Thorac. Surg..

[58] Badhwar V., Wei L.M., Cook C.C., Hayanga J.W.A., Daggubati R., Sengupta P.P., Rankin J.S. (2021). Robotic aortic valve replacement. J. Thorac. Cardiovasc. Surg..

[59] Sun J., Yuan Y., Song Y., Hu Y., Bai X., Chen J., Zhong Q. (2022). Early results of totally endoscopic robotic aortic valve replacement: Analysis of 4 cases. J. Cardiothorac. Surg..

[60] Hoffman J.I., Kaplan S. (2002). The incidence of congenital heart disease. J. Am. Coll. Cardiol..

[61] Stout K.K., Daniels C.J., Aboulhosn J.A., Bozkurt B., Broberg C.S., Colman J.M., Crumb S.R., Dearani J.A., Fuller S., Gurvitz M. (2019). 2018 AHA/ACC Guideline for the Management of Adults With Congenital Heart Disease: A Report of the American College of Cardiology/American Heart Association Task Force on Clinical Practice Guidelines. Circulation.

[62] Kodaira M., Kawamura A., Okamoto K., Kanazawa H., Minakata Y., Murata M., Shimizu H., Fukuda K. (2017). Comparison of Clinical Outcomes After Transcatheter vs. Minimally Invasive Cardiac Surgery Closure for Atrial Septal Defect. Circ. J..

[63] Balkhy H.H., Nisivaco S., Torregrossa G., Kitahara H., Patel B., Grady K., Coleman C. (2022). Multi-spectrum robotic cardiac surgery: Early outcomes. JTCVS Tech..

[64] Cerny S., Oosterlinck W., Onan B., Singh S., Segers P., Bolcal C., Alhan C., Navarra E., Pettinari M., Van Praet F. (2022). Corrigendum: Robotic Cardiac Surgery in Europe: Status 2020. Front. Cardiovasc. Med..

[65] Doulamis I.P., Spartalis E., Machairas N., Schizas D., Patsouras D., Spartalis M., Tsilimigras D.I., Moris D., Iliopoulos D.C., Tzani A. (2019). The role of robotics in cardiac surgery: A systematic review. J. Robot. Surg..

[66] Yanagawa F., Perez M., Bell T., Grim R., Martin J., Ahuja V. (2015). Critical Outcomes in Nonrobotic vs Robotic-Assisted Cardiac Surgery. JAMA Surg..

[67] Morgan J.A., Peacock J.C., Kohmoto T., Garrido M.J., Schanzer B.M., Kherani A.R., Vigilance D.W., Cheema F.H., Kaplan S., Smith C.R. (2004). Robotic techniques improve quality of life in patients undergoing atrial septal defect repair. Ann. Thorac. Surg..

[68] Deeba S., Aggarwal R., Sains P., Martin S., Athanasiou T., Casula R., Darzi A. (2006). Cardiac robotics: A review and St. Mary’s experience. Int. J. Med. Robot. Comput. Assist. Surg. MRCAS.

[69] Lewis C.T., Bethencourt D.M., Stephens R.L., Cline J.L., Tyndal C.M. (2014). Robotic repair of sinus venosus atrial septal defect with partial anomalous pulmonary venous return and persistent left superior vena cava. Innovations.

[70] Kadan M., Erol G., Kubat E., İnce M.E., Akyol F.B., Karabacak K., Doğancı S., Yıldırım V., Bolcal C., Demirkılıç U. (2022). Robotic repair of atrial septal defect with partial pulmonary venous return anomaly: Our 5 year experience. Int. J. Med. Robot. Comput. Assist. Surg. MRCAS.

[71] Sepúlveda E., Ibáñez A., Baeza C., Espíndola M., Sepúlveda G., Maureira M., Uribe J.P., Salas C. (2019). Robotic mitral valve repair and closure of atrial septal defect. Report of 13 procedures. Rev. Medica Chile.

[72] Thapmongkol S., Sayasathid J., Methrujpanont J., Namchaisiri J. (2012). Beating heart as an alternative for closure of secundum atrial septal defect. Asian Cardiovasc. Thorac. Ann..

[73] Cheng Y., Chen H., Mohl W., Liu X., Si Z. (2013). Totally endoscopic congenital heart surgery compared with the traditional heart operation in children. Wien. Klin. Wochenschr..

[74] Watanabe G., Ishikawa N. (2014). Alternative method for cardioplegia delivery during totally endoscopic robotic intracardiac surgery. Ann. Thorac. Surg..

[75] Yun T., Kim H., Sohn B., Chang H.W., Lim C., Park K.H. (2022). Robot-Assisted Repair of Atrial Septal Defect: A Comparison of Beating and Non-Beating Heart Surgery. J. Chest Surg..

[76] Gao C., Yang M., Wang G., Wang J., Xiao C., Wu Y., Li J. (2010). Totally endoscopic robotic atrial septal defect repair on the beating heart. Heart Surg. Forum.

[77] Zhe Z., Kun H., Xuezeng X., Yunge C., Zengshan M., Huiming G., Liming L., Liang T., Zhiwei W., Hansong S. (2014). Totally thoracoscopic versus open surgery for closure of atrial septal defect: Propensity-score matched comparison. Heart Surg. Forum.

[78] Kitahara H., Okamoto K., Kudo M., Yoshitake A., Ito T., Hayashi K., Inaba Y., Akamatsu Y., Shimizu H. (2016). Alternative peripheral perfusion strategies for safe cardiopulmonary bypass in atrial septal defect closure via a right minithoracotomy approach. Gen. Thorac. Cardiovasc. Surg..

[79] Harky A., Chaplin G., Chan J.S.K., Eriksen P., MacCarthy-Ofosu B., Theologou T., Muir A.D. (2020). The Future of Open Heart Surgery in the Era of Robotic and Minimal Surgical Interventions. Heart Lung Circ..

[80] Du Z.D., Hijazi Z.M., Kleinman C.S., Silverman N.H., Larntz K. (2002). Comparison between transcatheter and surgical closure of secundum atrial septal defect in children and adults: Results of a multicenter nonrandomized trial. J. Am. Coll. Cardiol..

[81] Crawford G.B., Brindis R.G., Krucoff M.W., Mansalis B.P., Carroll J.D. (2012). Percutaneous atrial septal occluder devices and cardiac erosion: A review of the literature. Catheter. Cardiovasc. Interv..

[82] Tchantchaleishvili V., Melvin A.L., Ling F.S., Knight P.A. (2014). Late erosion of Amplatzer septal occluder device resulting in cardiac tamponade. Interact. Cardiovasc. Thorac. Surg..

[83] Jalal Z., Hascoet S., Baruteau A.E., Iriart X., Kreitmann B., Boudjemline Y., Thambo J.B. (2016). Long-term Complications After Transcatheter Atrial Septal Defect Closure: A Review of the Medical Literature. Can. J. Cardiol..

[84] Kadirogullari E., Onan B., Timur B., Birant A., Reyhancan A., Basgoze S., Aydin U. (2020). Transcatheter closure vs totally endoscopic robotic surgery for atrial septal defect closure: A single-center experience. J. Card. Surg..

[85] Gao C., Yang M., Wang G., Xiao C., Wang J., Zhao Y. (2012). Totally endoscopic robotic ventricular septal defect repair in the adult. J. Thorac. Cardiovasc. Surg..

[86] Schilling J., Engel A.M., Hassan M., Smith J.M. (2012). Robotic excision of atrial myxoma. J. Card. Surg..

[87] Rodriguez E., Cook R.C., Chu M.W.A., Chitwood W.R. (2009). Minimally Invasive Bi-Atrial CryoMaze Operation for Atrial Fibrillation. Oper. Tech. Thorac. Cardiovasc. Surg..

[88] Medtronic Hugo. https://www.medtronic.com/covidien/en-us/robotic-assisted-surgery/hugo-ras-system.html.

[89] Johnson & Johnson Ottava. https://www.careers.jnj.com/robotics.

[90] CMR Surgical Versius. https://cmrsurgical.com/.

[91] Stryker MAKO SmartRobotics. https://www.stryker.com/pl/pl/index.html.

[92] Medicaroid Hinotori Surgical Robot System. https://www.medicaroid.com/en/.

[93] Titan Medical Enos 2.0. https://titanmedicalinc.com/.

[94] Moon Surgical Maestro system. https://www.moonsurgical.com/.

[95] Virtual Incision MIRA. https://virtualincision.com/.

[96] The Robot Report MIRA Surgical Robot to Be Tested in Space in 2024. https://www.therobotreport.com/virtual-incisions-mira-to-be-sent-to-the-iss-2024/.

[97] Nawrat Z., Krawczyk D. (2019–2020). Robin heart or how to overcome the distance and use a man as an element of the telemanipulator control system. Med. Robot. Rep..

[98] Pittsburgh Business Times Aethon Launches New Robot, Enters Hospitality Market. https://www.bizjournals.com/pittsburgh/news/2021/07/13/aethon-launches-new-robot-enters-new-market.html.

[99] Diligent Robotics Moxi. https://www.diligentrobots.com/moxi.

[100] Pudu Robotics. https://www.pudurobotics.com/.

[101] Robotarm My Spoon. https://robots.nu/en/robot/my-spoon.

[102] CareMeal Meal Assist Robot. https://www.iphoneness.com/smart-robots/caremeal/.

[103] Liftware Level. https://www.liftware.com/level/.

[104] Vitestro. https://vitestro.com/vitestro-unveils-autonomous-blood-drawing-device-combining-artificial-intelligence-ultrasound-imaging-and-robotics/.

[105] Leipheimer J.M., Balter M.L., Chen A.I., Pantin E.J., Davidovich A.E., Labazzo K.S., Yarmush M.L. (2019). First-in-human evaluation of a hand-held automated venipuncture device for rapid venous blood draws. Technology.

[106] iRobot. https://web.archive.org/web/20120103091646/http:/www.irobot.com/sp.cfm?pageid=203.

[107] Defi I.R., Iskandar S., Charismawati S., Turnip A., Novita D. (2022). Healthcare Workers’ Point of View on Medical Robotics During COVID-19 Pandemic—A Scoping Review. Int. J. Gen. Med..

[108] Five Critical Vulnerabilities Found in Aethon TUG Robots. https://heimdalsecurity.com/blog/aethon-tug-robots-have-been-found-to-have-critical-vulnerabilities/.

[109] Aethon TUG T2. https://aethon.com/PDF/TUGAccessedsheet.pdf.

[110] Aethon TUG T3. https://aethon.com/wp-content/uploads/2018/03/DatasheetT3_V3.pdf.

[111] Dinsaw. https://www.dinsaw.com/.

[112] Giraff Robot. https://telepresencerobots.com/robots/giraff-telepresence/.

[113] Grace Robot. https://edition.cnn.com/2021/08/19/asia/grace-hanson-robotics-android-nurse-hnk-spc-intl/.

[114] PARO Therapeutic Robot. http://www.parorobots.com/users.asp.

[115] Pepper Robot. https://support.unitedrobotics.group/en/support/solutions/articles/80000958735-pepper-technical-specifications.

[116] Vañó E., González L., Guibelalde E., Fernández J.M., Ten J.I. (1998). Radiation exposure to medical staff in interventional and cardiac radiology. Br. J. Radiol..

[117] Delichas M., Psarrakos K., Molyvda-Athanassopoulou E., Giannoglou G., Sioundas A., Hatziioannou K., Papanastassiou E. (2003). Radiation exposure to cardiologists performing interventional cardiology procedures. Eur. J. Radiol..

[118] Roguin A., Goldstein J., Bar O. (2012). Brain tumours among interventional cardiologists: A cause for alarm? Report of four new cases from two cities and a review of the literature. EuroIntervention.

[119] Vano E., Kleiman N.J., Duran A., Romano-Miller M., Rehani M.M. (2013). Radiation-associated lens opacities in catheterization personnel: Results of a survey and direct assessments. J. Vasc. Interv. Radiol. JVIR.

[120] Campbell P.T., Kruse K.R., Kroll C.R., Patterson J.Y., Esposito M.J. (2015). The impact of precise robotic lesion length measurement on stent length selection: Ramifications for stent savings. Cardiovasc. Revasc. Med. Incl. Mol. Interv..

[121] Bezerra H.G., Mehanna E., Vetrovec G.W., Costa M.A., Weisz G. (2015). Longitudinal Geographic Miss (LGM) in Robotic Assisted Versus Manual Percutaneous Coronary Interventions. J. Interv. Cardiol..

[122] Madder R.D., VanOosterhout S.M., Jacoby M.E., Collins J.S., Borgman A.S., Mulder A.N., Elmore M.A., Campbell J.L., McNamara R.F., Wohns D.H. (2017). Percutaneous coronary intervention using a combination of robotics and telecommunications by an operator in a separate physical location from the patient: An early exploration into the feasibility of telestenting (the REMOTE-PCI study). EuroIntervention.

[123] Bai R., Di Biase L., Valderrabano M., Lorgat F., Mlcochova H., Tilz R., Meyerfeldt U., Hranitzky P.M., Wazni O., Kanagaratnam P. (2012). Worldwide experience with the robotic navigation system in catheter ablation of atrial fibrillation: Methodology, efficacy and safety. J. Cardiovasc. Electrophysiol..

[124] Schachner T., Bonaros N., Wiedemann D., Weidinger F., Feuchtner G., Friedrich G., Laufer G., Bonatti J. (2009). Training surgeons to perform robotically assisted totally endoscopic coronary surgery. Ann. Thorac. Surg..

